# Same, Same But Different: How the Interplay of Legal Procedures and Structural Factors Can Influence the Use of Coercion

**DOI:** 10.3389/fpsyt.2019.00249

**Published:** 2019-04-24

**Authors:** Lieselotte Mahler, Juliane Mielau, Andreas Heinz, Alexandre Wullschleger

**Affiliations:** Charité–Universitätsmedizin zu Berlin, corporate member of Freie Universität Berlin, Humboldt-Universität zu Berlin, and Berlin Institute of Health, Berlin, Germany

**Keywords:** recovery-orientation, mutiprofessional decision-making processes, coercion, human rights, acute wards

## Introduction

Although clinicians and scientists have made persistent effort to reduce the use of coercive measures such as mechanical restraint, seclusion, and forced medication, it is required in some situations and staff members are thus confronted with a clinical and ethical dilemma: Coercive measures can save lives (e.g., when treating a Delirium Tremens) but can be linked with many negative consequences, ranging from a degradation of the therapeutic relationship to symptoms of posttraumatic stress disorder (1, 2). Moreover, the issue of perceived coercion has become a major concern over the past years. Patients’ feelings of not being respected and involved in decision-making processes can lead to higher levels of perceived coercion (3). Participation and freedom of choice regarding therapy and medication were described as highly relevant to patients (4).

Studies on the use of coercive measures indicate vast discrepancies between countries and institutions, therefore raising the question of factors influencing decision-making processes including legislative, institutional, and staff-related aspects (5–7). Authors underline the need to actively address ethical issues regarding the use of coercive measures as a tool to reduce their use to the absolute necessary minimum (8, 9).

The ratification of the UN Convention on the Rights of Persons with Disabilities (UN CRPD) has shed a new light on this matter and raised an important debate in the field of mental health (10). The Convention states that the presence of disability does not justify the application of compulsory treatments and that treatment decisions should, under any circumstances, respect the will and preferences of the persons with disabilities. These terms of “will and preferences” have been thoroughly discussed and defined by several authors (11); here, we refer to George Szmukler (12). He stresses that the application of compulsory treatment might only be justified if it aims at respecting a person’s will—defined as the expression of “deeply held, reasonably stable and reasonably coherent personal values”—and restoring the ability to express one’s will, in cases where this differs from the expressed preferences—defined as expressed “desires and inclinations” (12). The convention thus underlines that the patients’ perspective on their situation and treatment should always be actively assessed and integrated in the decision-making process regarding the use of coercive measures.

These ethical questions, along with the statements of the CRPD, urge psychiatric institutions to control their structures and treatment concepts in order to create the conditions needed to fulfill the afore-mentioned requirements (13–15).

In Germany, the highest court of justice, the Federal Constitutional Court, stated on the case of a forensic patient in 2011 that compulsory treatment can only take place with the intention of restoring the patients’ capacity to consent and only if several requisites are fulfilled. These encompass the impaired capacity of the patient to consent to the treatment after different options have been presented and explained, the necessary character of treatment to avoid acute endangerment of the patient or others, and the use of compulsory treatment as a last resort after all other alternatives have been exhausted.

These legally binding statements and the related discussions show that decision-making processes regarding the use of coercion need to be reviewed and revised accordingly. The interpretation of the preconditions for compulsory treatment, notably its “last resort” character, requires in-depth considerations.

Two short clinical cases from an acute psychiatric ward aim to highlight some of the core aspects of exemplary decision-making processes and underline the structural factors that these should be based on.

## Case Examples

Two patients were taken to an acute ward at the Department of Psychiatry of the St-Hedwig Hospital (Psychiatrische Universitätsklinik, Charité im St-Hedwig Krankenhaus) by police force in handcuffs on the same day within a 3-h period and were admitted to a general psychiatric ward on the legal basis of the Mental Health Law (Berliner PsychKG). Both patients were previously unknown to the police authority and the hospital staff and did not hold valid documents authorizing them. Their behavior attracted the attention of the police through imminent endangerment of others and “confusion.” To simplify the presentation of the different courses of treatment, we will refer to them as patients 1 and 2. All personal patient data have been modified to avoid their identification.

### Patient 1

Patient 1 (20–25 years old) presented with agitation. He was threatening, screaming, scratching, and spitting, and refused a conversation. He looked well-groomed (clothing, hair, dental status, cleanliness of skin and nails). The team, consisting of two nurses, a resident and a consultant psychiatrist, had the impression that the aggression of patient 1 was somehow undirected, i.e., not directed against certain persons and irrespective of the context. The perceived subjective and clinical aspects led to the assumption that patient 1 could suffer from an acute and potentially first manifestation of a mental disorder. He expressly refused to undergo medical examination and all offered treatments. The team tried many times to establish contact with the patient by calmly addressing him or offering him to sit down and talk, to drink something, or to retreat in a quiet room and rest. All of these attempts to de-escalate the situation didn’t have any effect. The patient was still agitated, threw himself against the ward door, thus bruising himself, or screamed at the staff. The team members thoroughly discussed the next steps to solve the acute situation.The involved staff members agreed that, in this situation, the legal conditions allowing the use of compulsory treatment and mechanical restraint were fulfilled and that, most importantly, every alternative had been exhausted. The team thus decided that, in order to prevent further harm to himself and others, compulsory treatment was the only available possibility. Because of the acute and dangerous character of the situation, the patient was then, according to the Mental Health Law, mechanically restraint, a blood analysis and an ECG were performed, and he received an i.v. medication. Legal procedures regarding the pursuit of the involuntary hospitalization and compulsory treatments including external medical review and a decision by a judge were initiated. The results of the analysis showed that the symptoms of patient 1 were caused by a severe overdose of L-thyroxine and an electrolyte imbalance due to anorexia nervosa. After a few days of intensive care treatment, patient l switched to outpatient treatment on another ward.

### Patient 2

Patient 2 (40–45 years old) presented with severe agitation. He was threatening, screaming, scratching, and spitting, and refused a conversation. He thus showed a similar clinical picture as patient 1 but also appeared to experience auditory hallucinations and to actively talk to them. Patient 2 was in a state of poor hygiene. Taking into consideration his manner of response, one could assume that patient 2 has experienced psychiatric treatment in the past. When the nurse asked him if he had any experience with psychiatric medication, he yelled at her and clarified his wish to refuse haloperidol. He seemed to feel especially threatened by the police and the psychiatric staff, not only due to psychotic symptoms but also due to previous aversive experiences with psychiatric treatment. Once again, the staff members involved in the situation discussed the clinical case in a multiprofessional setting and weighed out every possible option. The team suspected that patient 2 suffered from an acute exacerbation of a disorder that persisted for a longer period of time or a psychotic relapse. In this case, the team decided that patient 2—due to his previous aversive experiences—would have extraordinarily suffered from compulsory treatment, which may exacerbate previous traumatic experiences. Also, he calmed down a bit when given a space to withdraw and did not immediately endanger himself or others; however, he remained tense for several days and threw objects whenever members of staff tried to engage him in a conversation or offered oral medication. When left alone, he did not appear aggressive or present improper handling, showed a regular food intake, and welcomed the possibility to smoke. Somewhat later, he was seeking a medical consultation and expressed the need for a low-dosage medication. To this day, 6 years later, he regularly receives outpatient care and short-term crisis intervention treatment on a psychiatric ward, although he has felt threatened and deprived of his identity by the state and the psychiatric system of another city for more than 25 years.

## Discussion

These clinical cases elucidate the complexity of decision-making processes regarding the use of coercive measures such as mechanical restraint and forced medication. Both persons presented with impaired capacity to consent and acute endangerment of themselves or others. However, their situations differed with respect to their subjective reactions, previous experiences, and response to de-escalation. These factors played a central role in the evaluation of the possible alternatives and eventually in the whole decision-making process.

Decision-making processes leading to involuntary admission often imply uncertainty and doubt among the clinicians in charge (16). In a qualitative interview involving psychiatrists, Feiring and Ugstad showed that legal criteria regulating the use of compulsory admissions are often being interpreted by clinicians and that decisions are influenced by extra-legal factors, such as patients’ needs and attitudes toward treatment, follow-up options, and social circumstances (17). More precisely, the lack of less coercive alternatives has already been shown to be one of the major factors in the decision to commit patients against their will (18, 19). The issue of alternatives to coercive treatment has been addressed in different studies or scientific papers, confirming that patients place great emphasis on attention and consideration of their wishes and needs. In many cases, patients have the impression that the treating teams do not take their wishes into account (13). Many of them wish to get more support and contact during and after restraint, but also before the coercive event takes place in order to prevent it (20).

This suggests that the conditions facilitating the exploration of alternatives to coercion need to be elucidated (21, 22). The thorough search for alternative measures and their practical application require structures and attitudes within the team that facilitate the development of de-escalating competences of all professional groups and the acknowledgement of their subjective experiences. Accordingly, in the situation of patient 2, a coercive intervention could be avoided thanks to the clinical experience of the team, its orientation towards non-coercive treatment, and the possibility to provide the patient sufficient time and space.

Furthermore, the two cases suggest that in situations in which patients harm themselves and others, it is essential to comprehend the motivation behind aggressive or self-harming behavior. Whereas professional members of staff predominantly view the psychopathology as the key factor in the development of aggression, patients mostly believe that environmental factors (rules, communication style) play a crucial part (23). The interplay of psychopathology and external factors becomes apparent in the clinical case of patient 2: Although the potential danger of aggressive actions on the ward can be ascribed to psychotic symptoms, an escalation of physical violence based on former aversive experiences with the psychiatric system could and, in our opinion, would have been caused by restriction or other coercive measures. The link between a history of traumatic events and the experience of coercion has already been underlined in research works (24).

In recent years, complex interventions for acute psychiatric settings have been increasingly recommended to achieve a reduction of coercive interventions, as described in the “Weddinger Model”—a recovery-oriented treatment concept that has been introduced in the Charité University Department of Psychiatry at the St. Hedwig Hospital in 2010 and which aims to promote team competence on multiple levels (25–27). With regard to the outlined clinical cases, it should be elucidated that such interventions need to ensure an active participation of all team members in order to recognize their competences and collectively promote decision-making processes, as team dynamics seem to play a role in decision-making processes leading to coercion (28). Open settings can further reduce coercion (29). The promotion of knowledge about recovery and the appraisal of the therapeutic qualities of all professional groups on the ward are also part of the implemented model. In this regard, previous works argued that the staff attitude to coercion seems to be mainly linked to individual staff-related factors (30) and that an active effort into building trust in the therapeutic relationship can help improve it and prevent acute crisis situations (31).

In conclusion, it should be noted that a reduction of coercion in psychiatric settings appears promising if legal procedures and oversight are combined with multiprofessional, patient-centred, and recovery-oriented clinical work relevant to the complexity of any acute crisis situation.

## Author Contributions

LM, JM, AH, and AW conceived and wrote the paper.

## Conflict of Interest Statement

The authors declare that the research was conducted in the absence of any commercial or financial relationships that could be construed as a potential conflict of interest.
